# When Dark Humor and Moral Judgment Meet in Sacrificial Dilemmas: Preliminary Evidence With Females

**DOI:** 10.5964/ejop.2417

**Published:** 2021-11-30

**Authors:** Emmanuelle Brigaud, Nathalie Blanc

**Affiliations:** 1 Department of Psychology, University of Paul Valéry Montpellier 3, Montpellier, France; University of Liverpool, Liverpool, United Kingdom

**Keywords:** moral judgment, sacrificial dilemmas, dark and nondark humor

## Abstract

The influence of dark humor on moral judgment has never been explored, even though this form of humor is well-known to push the boundaries of social norms. In the present study, we examined whether the presence of dark humor leads female participants to approve a utilitarian response (i.e., to kill one to save many) in sacrificial dilemmas. The effects of two types of humorous contexts were compared (i.e., dark vs. nondark) on dilemmas, which differed according to whom benefits from the crime (i.e., oneself and others vs. others only). In addition to collecting moral responses, individuals’ emotional states were assessed at three critical steps: Before and after reading the jokes and also after performing the moral judgment task. Our results revealed that dark and nondark humor similarly elicited a positive emotional state. However, dark humor increased the permissiveness of the moral violation when this violation created benefits for oneself. In self and other beneficial dilemmas, female participants in the dark humorous condition judged the utilitarian response more appropriate than those in the nondark condition. This study represents a first attempt in deepening our understanding of the context-dependent nature of moral judgment usually assessed in sacrificial dilemmas.

Suppose a runaway trolley is about to run over and kill five people. Suppose further that a large stranger is standing on a bridge over the tracks and that the only way to stop the trolley is to push that person in front of the trolley, killing him for sure but saving the others. Would it be okay to sacrifice one life to save several others? Most people answer “no” to this “high conflict” personal moral dilemma (Greene et al., 2001, 2004).

This phenomenon has been widely studied by psychologists to understand the cognitive and affective processes underlying moral judgments (see Waldmann et al., 2012 for a review; see also Bartels et al., 2015). The dual-process theory provides a relevant framework to explain people’s responses to sacrificial dilemmas (Greene, 2007; Greene et al., 2001, 2004, 2008). According to this well-known theory, two separate systems are involved in moral judgment: the controlled cognitive process, which corresponds to conscious reasoning (slow and effortful), and the automatic emotional one based on intuition and affects (fast and largely unconscious). In response to “high conflict” personal dilemmas, like the footbridge scenario described above (Thomson, 1985), people are typically driven by automatic emotional responses and judge that it is morally unacceptable to push someone off a footbridge even though not pushing him would result in a greater number of deaths. The perspective entailing a moral violation, such as killing an innocent person triggers a strong emotional aversion that inhibits an amoral solution (Greene, 2008). However, with sufficient time, motivation or resource conditions, people may engage in controlled cognitive processes regarding the costs and the benefits of killing another person. Such mechanisms result in a utilitarian judgment: approving the sacrifice of one life in order to save the lives of five is morally acceptable with respect to the number of victims.

In a footbridge-type dilemma, the rational choice (i.e., sacrifice the life of one person in order to save the lives of a greater number of people) is directly in conflict with deontological rules or intuition. Consequently, the utilitarian response requires one to control or overcome the initial aversive reaction against harming an innocent person (Greene, 2008). Two sets of studies have provided converging evidence in line with this idea. First, both empirical and neuropsychological studies have shown that utilitarian judgments are due to an absent or a reduced affective response. Participants with emotion-related neurological deficits (e.g., Ciaramelli et al., 2007; Koenigs et al., 2007; Moretto et al., 2009), with decreased empathic concern or with antisocial personality traits (Bartels & Pizarro, 2011; Conway & Gawronski, 2013; Gleichgerrcht & Young, 2013; Kahane et al., 2015) reach more utilitarian conclusions in sacrificial dilemmas. Secondly, other researchers have shown a link between the utilitarian response and working memory capacity (Moore et al., 2008) and also between this type of response and an individuals’ need for cognition (Bartels, 2008; Conway & Gawronski, 2013). Precisely, participants are more likely to approve a utilitarian response when they scored high in working memory capacity or in need for cognition (a motivational tendency to seek and enjoy effortful cognitive activity).

Concomitantly, environment-induced positive mood at the time of judgment increases a utilitarian response. For instance, simple exposure to humorous material before the presentation of the footbridge scenario increases permissiveness for moral violations (i.e., pushing the stranger over the bridge; Strohminger et al., 2011; Valdesolo & DeSteno, 2006). Such effect arises, because humor is usually associated with the experience of a positive emotion (i.e., mirth, laughter, pleasure). Therefore, if humor induces positive emotion at the time of judgment, the perceived negativity linked to any potential moral violation is attenuated and, thus, utilitarian response increases. This explanation is consistent with Fredrickson’s (2001) hypothesis that positive emotions can act as an antidote to negative emotions correcting or diminishing their influence.

Humor, used as a communicative activity, elicits positive emotional reactions in perceivers and tendency to laugh (Gervais & Wilson, 2005; Martin & Ford, 2018; Veatch, 1998). It also indicates to the target or audience that what happening, or is going to happen, should be taken as a joke (Gervais & Wilson, 2005; Ramachandran, 1998). In Ford's et al. (2008) words: “humor invokes a conversational rule of levity, that is, humor communicates an implicit message to the receiver that the usual rules of logic and expectations of common sense did not apply” (p. 160). In the context of social judgment, this central property of humor might allow us to treat the violation of moral rules (e.g., “it’s forbidden to kill”) as a matter of play and, therefore, favor the utilitarian judgment. Consistent with this hypothesis, Strohminger et al. (2011) found that mirth (i.e., the positive emotion associated with humor) increases permissiveness for deontological violation in moral dilemmas, whereas elevation (i.e., a positive emotion experienced upon witnessing another person perform a virtuous act; Algoe & Haidt, 2009; Haidt, 2003) has the opposite effect. This result highlighted that the influence of humor on people’s moral judgment cannot be explained simply in terms of experiencing positive emotions. They suggest that humor influences moral judgment by removing the gravitas of the moral violation (i.e., making immoral behavior funny). This interpretation is consistent with the Benign Violation Theory of humor (McGraw & Warren, 2010; McGraw et al., 2012; Veatch, 1998), which suggests that humor occurs when people simultaneously appraise a violation as being normal, acceptable, or okay.

The aim of the present study was to investigate more thoroughly to what extent a humorous context can influence the response of participants in personal moral dilemmas. The only two studies (Valdesolo & DeSteno, 2006; Strohminger et al., 2011) that focused on the role of humor on moral judgment used non-transgressive humor (i.e., inoffensive comedy). Thus, it would be interesting to see if the observed humor effect on moral judgment could be stronger when one uses humor with a transgressive content. As this form of humor is closely linked to the transgression of social norms and moral systems, it creates a context that could lead individuals to consider the utilitarian response in sacrificial dilemmas as harmless or okay. McGraw and Warren (2010) showed that moral violation is benign when another norm suggests that the behavior is acceptable or correct. In this sense, expression of transgression delivered in a humorous form could suggest that, in this context, the moral violation is okay (i.e., it’s acceptable to kill someone). This is closely linked with recent research that has shown that exposure to humorous forms of disparagement (i.e., sexist, racist or anti-gay jokes) lead to an increase in expression of prejudice toward target groups (e.g., O’Connor et al., 2017; Saucier et al., 2016; Woodzicka & Ford, 2010). According to the benign-violation theory hypothesis, such effects may occur because in the humorous context, the moral violation (i.e., denigrate a social group) is perceived as benign (see Gutiérrez et al., 2018; Thai et al., 2019, for a similar interpretation).

A particularly interesting form of humor to study in sacrificial dilemmas is dark humor because it treats sinister and tragic subjects, like death, with amusement and trivializes the victim’s suffering (Aillaud & Piolat, 2012). Dark humor (death-related humor) is described as cynical, gallows, morbid. Playing with serious or sad real life events, dark humor is generally considered as transgressive since it crosses the red line of social norms and moral systems. This form of humor takes its name from jokes about condemned men or hopeless victims to relieve tension before being executed (Freud, 1905). Thus, the condemned to death may well declare when led to the scaffold on a beautiful Monday morning, “This is a week that is starting well!” The juxtaposition of morbid and farcical elements in dark humor elicits two simultaneous perceptions: one, that the dark content constitutes a moral violation in which negative serious topics are mocked and, the other, an interpretation that the dark content is benign. Since dark humor treats negative serious ideas (like death, suffering of the victims or body integrity) in a light-hearted, playful manner (Charaudeau, 2006), it is not surprising that people use it as a coping strategy in stressful life-and-death situations (Christopher, 2015; Rowe & Regehr, 2010; van Wormer & Boes, 1997; Young, 1995). In the context of social judgment, dark humor might signal that the violation of moral rule (i.e., to kill someone) is fine and, therefore favor the utilitarian response. Thus, we predicted that participants exposed to dark humor before performing a moral judgment task would answer in a more utilitarian fashion (i.e., approving killing one) compared to participants exposed to nondark humor.

To deepen our understanding of moral judgment in a humorous context, we questioned whether the humor effect depends on who benefits from the crime. Our research considered cases where crime benefits oneself and others versus others only. We predicted that dark humor reinforces the tendency to morally approve the act of killing someone in particular when such action benefits the self in addition to others. This prediction is consistent with two results in psychology of morality (for a review see Ellemers et al., 2019). The first one showed that the tendency to produce utilitarian responses is strongly tied to consideration of self-interest (Christensen et al., 2014; Kahane et al., 2015). Indeed, people are more inclined to approve harm if their own life is at stake than if the moral transgression is merely to save others. The second one suggested that people feel less negative emotions (e.g., guilt and shame) about their dishonest actions and perceived them to be morally acceptable when there are other beneficiaries for these actions in addition to themselves (Gino et al., 2013). In this case, people use the potential benefits for others as a way to justify their self-interested and unethical actions (self-serving altruism). After being exposed to dark humor, committing harm could be considered less socially inappropriate when participants are faced with self and other beneficial dilemmas, because the crime relies on their own utility since this “selfish” consideration enables them to save others too.

The objective of the present research was to investigate more specifically the impact of two humorous contexts (i.e., dark and nondark jokes) on people’s responses to sacrificial dilemmas) as a function of whom benefits from the crime (other vs. self and other).

## Method

### Participants

One hundred and thirty-six female undergraduate students^1^1The original sample was composed of 136 female and 19 male participants (which is a classical sample of students enrolled in psychology courses). Because there are gender differences in the perception and the attitude toward dark and nondark humor (e.g., Aillaud & Piolat, 2012, 2014), we selected female participants only for this study. from the University of Montpellier 3 (France) took part in this experiment. The average age of the sample was 20.75 years (*SD* = 3.40). Informed consent was obtained from all students prior to participating in any of the tasks. They were informed that their responses remained anonymous in respect of the Data Protection law. All students received course credit as compensation.

### Materials

#### Humorous Materials

To assess the effects of humor on moral judgment, we used 12 jokes: six jokes were not transgressive (i.e., nonsense or clownish humor) and six dark jokes with a transgressive content (i.e., dealing with sinister topics with amusement like death, suffering of the victims and body integrity). For example, one of the dark jokes used:

A lawyer goes to the coroner about an autopsy:

– Before signing the death certificate, did you take this man’s pulse?

– No.

– Did you check to see if his heart was still beating?

– No.

– Did you check whether he was still breathing?

– No.

– So you signed this death certificate without performing any of the recommended tests for establishing whether a person is really and truly dead?

– Yes. Why? Did you find his head?

These jokes were selected on the basis of pretest ratings given by a total of 180 undergraduate students from the University of Montpellier 3, France (*M*_age_ = 19.84 years, *SD* = 2.63; the majority were female, 86%). They were all volunteers and were compensated with course credit for their participation.

A first group of 90 participants were asked to rate 30 jokes regarding their darkness. They rated “How dark is the joke?” using a scale from 1 (*not at all dark*) to 5 (*very dark*). Because participants were tested collectively, jokes were presented in a counterbalanced order across participants. Based on the results of this pilot study, we selected 12 jokes from the pool of the 30 rated jokes (i.e., those that lead to the most consistent appraisal among the sample): six jokes were attributed the lowest score (i.e., 1 = *not at all dark*) by at least 78% of the sample and six jokes were attributed the highest score (i.e., 5 = *very dark*) by at least 72% of the sample. All other jokes were excluded from the experimental material.

To ensure that this set of jokes (i.e., dark and nondark jokes) was similar in terms of funniness ratings, but distinct in terms of transgressive content, we recruited a second group of 90 participants. After reading each joke, they answered the two following questions: “How funny is the situation described in this joke?” and “How unbecoming and unseemly^2^2Indecent, inappropriate. is the situation described in this joke?.” Using the same procedure as Aillaud and Piolat (2012, 2014), responses were made on a 4-point scale (1 = *definitely not*, 2 = *not*, 3 = *slightly yes*, and 4 = *definitely yes*). Note that this 4-point scale enabled us to avoid a midpoint evaluation.

A two-way analysis of variance (ANOVA; Type of humor: Dark vs. Nondark) was made for each rating. These analyses revealed a main effect of transgressive content ratings only, *F*(1, 88) = 77.45, *p* < .001, $\eta_{p}^{2}$ = .47, dark jokes being judged as more unbecoming and unseemly (*M* = 2.64, *SD* = 0.69) than nondark ones (*M* = 1.23, *SD* = 0.32). There were no significant differences between dark and nondark jokes regarding funniness (*M* = 2.57, *SD* = 0.48 and *M* = 2.69, *SD* = 0.63, respectively), *F*(1, 88) = 0.93, *p* = .34. These results confirm that participants perceived a difference between dark and nondark humor solely on the transgressive dimension.

#### Moral Dilemmas

We selected four high conflict personal dilemmas from a previously used set (see Greene et al., 2001, 2004) in which the participant was always presented as the main protagonist of the situation (i.e., the one who was supposed to carry out the moral violation). The dilemmas were similar regarding at least two dimensions: All dilemmas involved killing one person in order to save several others; the number of people saved was comparable (*N* = 10). In addition, all these dilemmas were known to elicit mainly the same negative emotion (i.e., guilt) during judgment (Choe & Min, 2011). The dilemmas were only distinguished according to whom benefits (other vs. self and other) from the crime. The footbridge and the vitamins were the two other-beneficial dilemmas, while the lifeboat and safari were self and other beneficial ones. In the latter, the crime enabled one to save others as well as the protagonist herself. For example, in the lifeboat dilemma, the protagonist must choose whether to throw a person overboard to save the life of remaining passengers and her life too.

#### Emotional Scales

**Figure 1:**
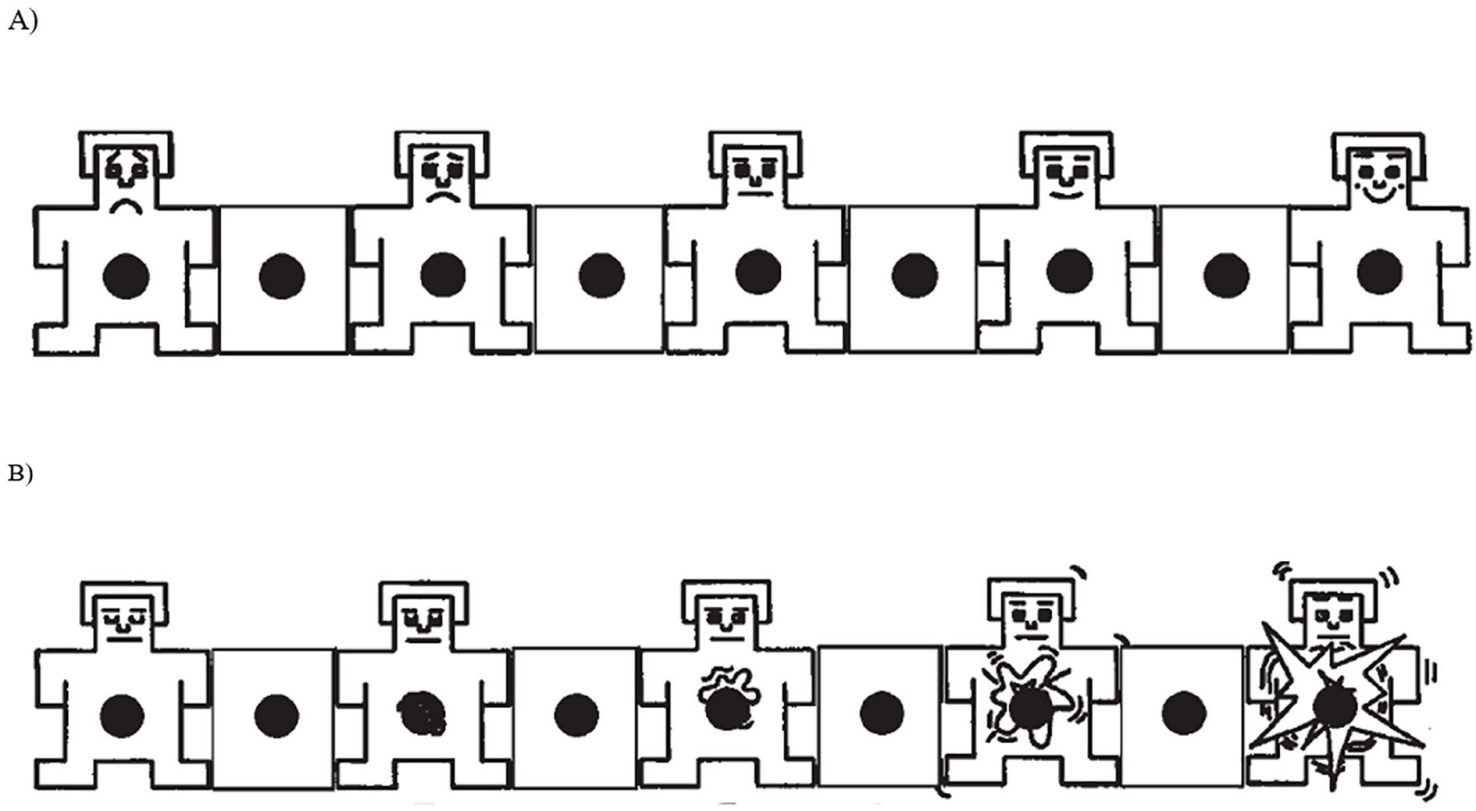
The SAM Scales for Valence (A) and Arousal (B) With the Five Figures and the Spaces Between Them Corresponding to One Point of the 9-Point Scale (Lang, 1980; see also Lang et al., 1997)

Participant’s emotional state was assessed on two dimensions: valence (positive vs. negative) and arousal (level of activation) using the Valence and the Arousal scales of the Self-Assessment Manikin (SAM; Lang, 1980). According to Bynion and Feldner (2017), the SAM is a brief and nonverbal measure of emotional state which reliability has been confirmed by numerous studies conducted in various domains (e.g., psychology, communication, advertising; Morris, 1995) and populations (e.g., gender, age, race; Backs et al., 2005; Nabizadeh Chianeh et al., 2012). The SAM scales consist of two sets of five figures depicting different levels of affective valence and arousal (see Figure 1). For each dimension, participants were instructed to place an “X” on or between the figures that best described their emotional state. The Valence scale (A) ranged from *unhappy* (1) to *happy* (9) and the Arousal scale (B) from *calm* (1) to *excited* (9).

### Procedure

After giving their informed consent, participants were randomly assigned either to the dark humor condition (*N* = 68) or to the nondark humor condition (*N* = 68). All participants were asked to complete an online questionnaire composed of two parts: First, they were exposed to six jokes and then they had to complete a moral judgment task. Immediately after reading the humorous material, all participants responded to four high-conflict personal dilemmas. The order of presentation of the dilemmas was counterbalanced within and between the dark and nondark humor conditions. For each dilemma, participants had to decide whether the utilitarian option (i.e., to kill someone) was appropriate or not (yes/no question). The answer “yes” always represented the utilitarian response. The scenarios were briefly introduced by stating that they refer to serious situations that could be seen as unpleasant but require making a difficult choice. To put the participants “in context” for the task that awaited them, and to ensure that they were engaged in the moral issues at stake, they were instructed to imagine themselves in each situation so that their answer could mirror their action in real life (e.g., see Tassy et al., 2013). They were asked to be as honest as possible in their responses, knowing that there is no good or wrong answer. In addition to recording responses to the dilemmas, we also assessed the participant’s emotional state in three steps: before reading the jokes (Time 1), after reading the jokes (Time 2) and after the moral judgment task (Time 3). Participants rated their emotional state using the Valence and Arousal scales of the SAM (Lang, 1980).

## Results

### Emotional States

To examine whether participants’ emotional states fluctuated throughout the experiment, a repeated ANOVA was conducted, first on the valence ratings and, second on arousal ratings. The type of humor (i.e., Nondark humor vs. Dark humor) was the between-participant factor, and the mood assessment time (Time 1 vs. Time 2 vs. Time 3) was the within-participant factor. Mean ratings (and standard deviation) of valence and arousal are reported in Table 1.

**Table 1:** Mean Ratings (and Standard Deviations) of Valence and Arousal for the Dark Humor and the Nondark Humor Conditions at Each of the Three Assessment Times

Type of humor	Valence ratings						Arousal ratings					
	Time 1		Time 2		Time 3		Time 1		Time 2		Time 3	
	*M*	*SD*	*M*	*SD*	*M*	*SD*	*M*	*SD*	*M*	*SD*	*M*	*SD*
Nondark humor	5.37	1.88	6.13	1.65	4.06	2.04	4.75	1.82	5.19	1.85	5.51	2.09
Dark humor	5.50	1.76	5.97	1.71	4.28	1.80	5.06	1.91	5.19	1.97	5.47	2.06

*Note.* Before (Time 1) and after (Time 2) reading the jokes; after the moral judgment task (Time 3).

Regarding valence ratings, a significant effect of time assessment was observed, *F*(2, 268) = 59.34, *p* < .001, $\eta_{p}^{2}$ = .31. Post hoc analysis (Scheffé test) revealed that participants reported feeling happier after reading the jokes than before reading the jokes (Time 2, *M* = 6.05, *SD* = 1.68; Time 1, *M* = 5.43, *SD* = 1.82), but their induced happiness decreased after the moral judgment task (Time 3, *M* = 4.17, *SD* =1.92) (*ps* < .001). Regarding arousal ratings, ANOVA revealed a significant effect of time assessment, *F*(2, 268) = 5.51, *p* < .01, $\eta_{p}^{2}$ = .04. Participants reported feeling more excited after (Time 3, *M* = 5.49, *SD* = 2.07) than before the experiment (Time 1, *M* = 4.90, *SD* = 1.86), (*p* < .01). No other effects were significant.

### Moral Judgment

**Figure 2:**
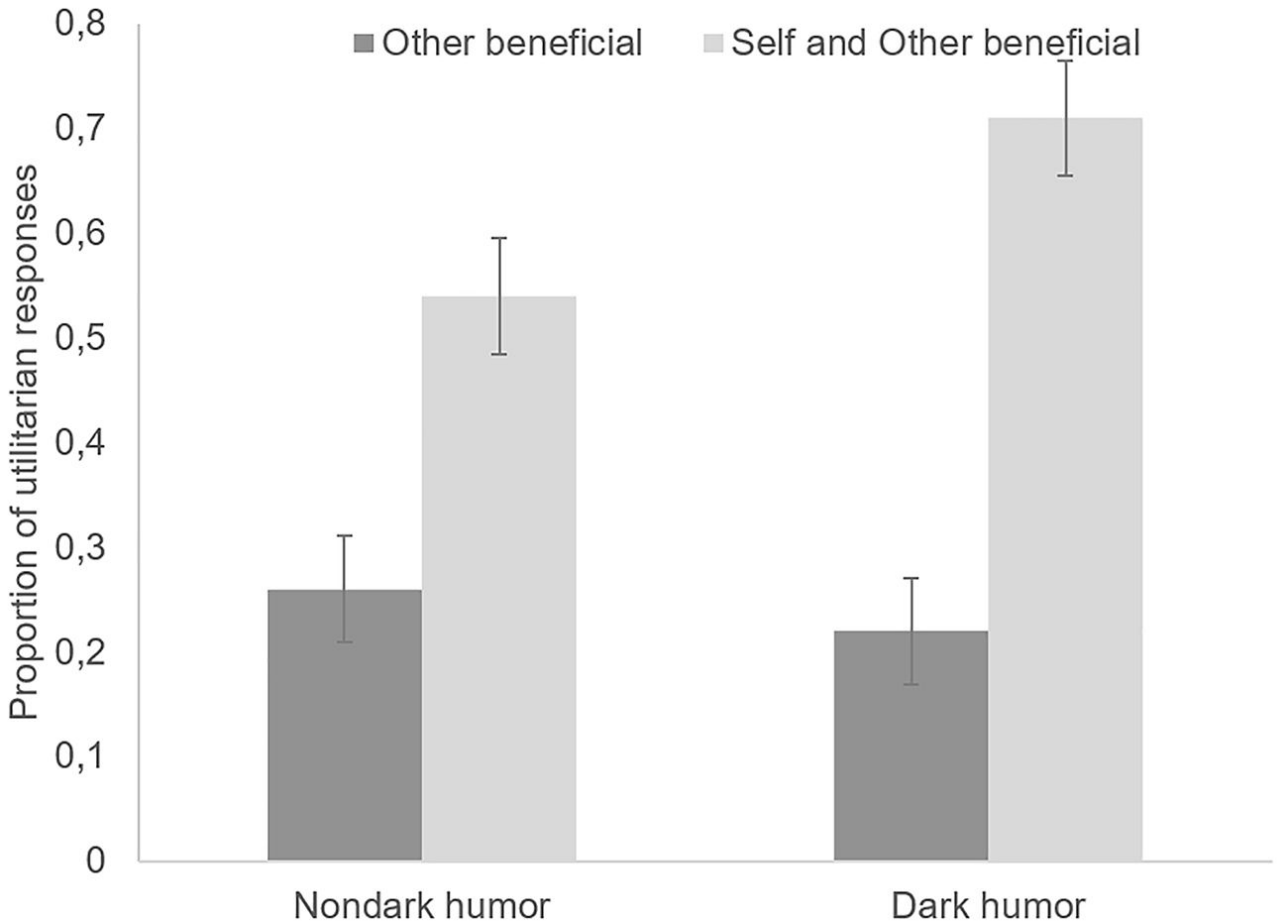
Mean Proportion of Utilitarian Responses as a Function of Type of Humor (Dark vs. Nondark) and Type of Dilemma (Self and Other Beneficial vs. Other Beneficial) *Note*. Error bars depict standard errors.

The mean proportion of utilitarian responses (i.e., killing is judged morally appropriate) was analyzed (ANOVA) to explore the effect of both the type of humor (i.e., Nondark humor vs. Dark humor) and the type of dilemma (i.e., Self and Other-beneficial vs. Other- beneficial). This 2 × 2 analysis showed a main effect of the type of dilemma indicating that killing to save oneself and others was judged to be more appropriate (*M* = 0.55, *SD* = 0.38) than killing to save only others (*M* = 0.19, *SD* = 0.30), *F*(1, 134) = 94.99, *p* < .001, $\eta_{p}^{2}$ = .41. A significant Type of humor × Type of dilemma interaction showed that this tendency to accept moral violation in their own self-interest increased when participants were exposed to dark jokes, *F*(1, 134) = 7.75, *p* = .006, $\eta_{p}^{2}$ = .05 (see Figure 2). Post hoc analysis (Scheffé test) revealed that, in self and other beneficial dilemmas, the mean proportion of utilitarian responses was significantly higher in the dark humor condition than in the nondark ones (*p* < .001). No significant difference between these two conditions was found for other-beneficial dilemmas (*p* = .60). No other effects were significant.

## Discussion

Compared to the existing literature dealing with humor and moral judgment (Strohminger et al., 2011; Valdesolo & DeSteno, 2006), the present study highlights the relevance of distinguishing different types of humorous inducing materials. Contrary to previous studies that only considered nondark humor, our comparison between dark and nondark humor revealed that variability in moral judgment could not simply be explained in terms of experiencing positive emotions or in terms of the levity property of humor. The content of humorous jokes deserves to be considered especially when this content relies on the transgression of social norms. Under the veil of amusement, moral violation in self and other beneficial scenarios increased in a more important way when participants were exposed to dark humor. To deepen our understanding of the underlying mechanisms of the permissiveness within different humorous contexts, four main lines of research must be considered.

First, it’s noteworthy that people are motivated by their self-interest and prone to behave for their own benefit in moral dilemmas (Christensen et al., 2014; Moore et al., 2008, 2011). In our study, individuals tended to exhibit a utilitarian response style when the transgressive act was described as self and other beneficial as compared to other-beneficial. This effect is coherent with Greene’s dual-process model (Greene et al., 2001, 2004, 2008), which suggests that utilitarian judgments result from a deliberate analysis of costs and benefits. In this cost-benefit perspective of moral judgment, saving oneself (in addition to others) could be considered as an additional benefit: The gains represented by saving oneself and others outweigh the gains of saving others only. Hence, people probably experience less conflict in the analysis of cost-benefit ratio when action is for their own benefit too. This interpretation is compatible with Moore et al. (2008, 2011) who showed that individuals were faster to approve the “utilitarian” response when those who benefited from the crime included themselves. This interpretation is also consistent with Shalvi et al. (2015; see also Gino et al., 2013) who underlined that people experience less internal conflicts when the temptation to profit from unethical behavior can be justified by saving others. This self-serving altruism could explain our results. In the present study, the self and other beneficial scenario enabled people to violate moral rules (e.g., approve a behavior that cause harm to a victim) while maintaining their positive self-image, because the moral violation also benefitted others.

Secondly, our results showed that the tendency to accept moral violation in both their self and other interest increased when participants were exposed to dark jokes. According to the benign violations theory literature (McGraw & Warren, 2010; Warren & McGraw, 2015, 2016), this result suggests that humorous contexts affect moral judgment via appraisal processes. Indeed, the fact that the permissiveness of the moral violation increases in self and other beneficial dilemmas under dark humorous context suggests that this informational context promotes the appraisal of utilitarian response as a benign violation. In other words, when negative serious ideas (like death or suffering of the victims) are associated to farcical elements through dark jokes, the utilitarian response probably becomes more benign, especially when it benefits oneself in addition to others. However, such explanation should be considered with caution since we did not directly emphasize the moral component of the utilitarian response. In the present study, the harmful actions described in sacrificial dilemmas were judged appropriate or inappropriate only. Future studies should directly investigate if utilitarian judgment is perceived as more or less immoral according to the type of humor (Dark vs. Nondark) and the type of dilemma (Self and Other-beneficial vs. Other- beneficial). Another limitation, inherent to almost all moral dilemma research is about the nature of the task and the corresponding measure. As highlighted by Crone and Laham (2017), sacrificial dilemma responses have to be considered with caution since they have been proved to be a poor indicator of moral values. Obviously, there is a huge difference between what one judges as morally acceptable and what one actually does (see also Tassy et al., 2013). A profitable line of research would consist of distinguishing between evaluative judgments and choices of action.

Thirdly, the way the scenarios are perceived is another line of research that deserves to be considered. Bauman et al. (2014) strongly recommend that researchers be cautious when using sacrificial dilemmas to studying moral judgment: The ecological validity of sacrificial dilemmas needs to be carefully considered (see Körner et al., 2019) because the lack of realism may threaten the validity of moral decision processes under interest. Also, because the scenarios are hypothetical, a utilitarian response (i.e., kill someone) could be seen as both a violation and benign. In this circumstance, the benign-violation predicts that people will be amused. In accordance with this hypothesis, Bauman et al. (2014) showed that, in the footbridge scenario, people considered pushing the man to be wrong, but also reported laughing. If sacrificial dilemmas have the power to elicit humor, we can hypothesize that exposure to dark jokes promotes the violation and benign appraisals of the situation described in the scenario, and thus, generates laughter. The question is can dark humor transform a serious scenario into a humorous one, because its transgressive content leads to perceive that moral violation is actually okay. Rather than abandoning sacrificial dilemmas entirely, using a virtual reality paradigm may offer a more vivid experience of the scenarios, making their realism more salient (e.g., McDonald et al., 2017; Slater & Sanchez-Vives, 2016), and elicit more serious moral deliberation.

A last but not least contribution of the present study concerns individuals’ emotional state when faced with moral dilemma. Contrary to previous studies that only considered nondark humor, our comparison between dark and nondark humor revealed that variability in moral judgment could not simply be explained in terms of experiencing positive emotions. Interestingly, the benign-violation theory (McGraw & Warren, 2010) suggests that humor does not systematically involve positive emotions (e.g., amusement, mirth). Because humor results from violations that are simultaneously seen as benign, it may elicit mixed emotions. This idea is in line with theorists (e.g., Larsen & McGraw, 2014; Larsen et al., 2001; Schimmack, 2001) who argue that positive and negative emotions do not mutually inhibit each other, and may at times even co-occur (i.e., mix). Clearly, humor elicits mixed feelings such disgust and amusement. For instance, people are both amused and disgusted when exposed to a disgusting humorous clip (Hemenover & Schimmack, 2007). Aillaud and Piolat (2014) provided additional evidence when underlying that participants used both positive and negative emotional lexicon to describe the emotional experience associated with dark and nondark humorous cartoons. These authors reported that dark humor is particularly conducive to provoking mixed emotions. Not only does its transgressive content elicit amusement, but also triggers negative emotions such as shame or disgust. Since the present study operationalized dark humor, participants may have felt amusement and shame (or/and disgust), two emotions of opposite valence. This hypothesis cannot be tested in our study since we measured emotional valence only. Our results revealed that participants reported feeling happier after reading the jokes than before reading the jokes, but their induced happiness decreased after the moral judgment task. Further research should consider the panel of discrete emotions to understand how individuals manage mixed feelings when asked to judge whether the acts are morally appropriate or not. It would be interesting to examine what they feel in the different steps: before the moral judgment task, during the reading of the scenario and after the moral judgment. Because the dilemmas have proven to elicit different negative emotions (i.e., guilt, disgust, sadness, empathy, anger; see Choe & Min, 2011), the question remains on how different types of humor can counterbalance such negative feelings.

Overall, there is no doubt that the next step to overcome in deepening our understanding of moral judgments is to focus on its context-dependent nature. This line of research allows us to get a better understanding of the mechanisms in which humor influences moral judgment. Some additional factors may contribute to this line of research like an individuals’ need for humor (Cline et al., 2003; see also Picard & Blanc, 2013) and also gender (e.g., Herzog & Anderson, 2000). Interestingly, in the present research, dark humor effects are observed on a sample composed exclusively of females. It is noteworthy that females are known to usually find less humor in dark events than males (Aillaud & Piolat, 2012). The tendency to produce a utilitarian response could be strongly reinforced under dark humor with males who are predisposed to generate and seek out humor (i.e., who scored high in need for humor). Future research is needed to test this hypothesis. Finally, this study sheds light on the necessity to not neglect the fact that moral judgments take place in a specific sociocultural environment more or less prompt to accept dark humor. The exposure to dark humor in an individualist culture is of great importance since moral decision experienced in everyday life is often driven by selfish attitudes. The presence of dark humor can promote moral transgression that favors the tolerance of utilitarian response.
